# Mental health problems and psychological burnout in Medical Health Practitioners: A study of associations and triadic comorbidity

**DOI:** 10.12669/pjms.35.6.444

**Published:** 2019

**Authors:** Farzana Ashraf, Hassaan Ahmad, Muneeba Shakeel, Sana Aftab, Afsheen Masood

**Affiliations:** 1 Dr. Farzana Ashraf, Department of Humanities, COMSATS University Islamabad, Lahore Campus, Pakistan; 2 Dr. Hassaan Ahmad, Department of General Surgery, Pakistan Institute of Medical Sciences (PIMS), Islamabad, Pakistan; 3 Dr. Muneeba Shakeel, Department of Humanities, COMSATS University Islamabad, Lahore Campus, Pakistan; 4 Dr. Sana Aftab, Department of Pediatrics, Pakistan Institute of Medical Sciences (PIMS), Islamabad, Pakistan; 5 Dr. Afhseen Masood, Institute of Applied Psychology, University of the Punjab, Lahore, Pakistan

**Keywords:** Depression, Anxiety, Stress, Psychological burnout

## Abstract

**Background and Objective:**

Mental health problems not only affect the common person but also medical health practitioners (MHPs) dealing with health care issues of patients. The current study aimed to explore the triadic (three dimensional) comorbidity of mental health problems and its association with three forms of psychological burnout (person, client and work related).

**Methods:**

This correlational study was conducted at three hospitals of Islamabad/ Rawalpindi (Holy Family Hospital=57, District Headquarter Hospital=60, and Benazir Bhutto Hospital=40) from June 2018 to September 2018. The sample comprised of 157 medical health practitioners (medical doctors) serving in general ward (n=64), emergency (n=60) and OPD (n=33) sections. The participants were administered self-report measures of DASS-21 and Copenhagen Burnout Inventory.

**Results:**

Triadic comorbidity of depression, anxiety and stress ranged from 9% to 26% for excessive severe and normal levels respectively. Comorbidity of work related and personal related burnout at severe level was found 8%. Further, depression, anxiety and stress symptoms were strongly associated with psychological burnout at .*0001* significance level.

**Conclusion:**

MHPs demonstrated excessive severe comorbidity of depression, anxiety and stress simultaneously. Along with this triadic comorbidity, the presence of severe psychological burnout is alarming and affecting overall efficiency and mental health of doctors which need to be identified, screened out and managed timely and managed properly.

## INTRODUCTION

Medicine being a very demanding profession is known to be highly stressful.^1^ Medical health practitioners (MHPs) are the individuals who provide preventive, curative, promotional or rehabilitative health care services in a very systematic fashion. Due to vast exposure of health related issues and psychologically challenged circumstances of patients, MHPs are also likely to induce health related symptoms particularly in the context of mental health which is generally defined by presence of depression, anxiety and stress symptoms generally manifested as sadness, low mood, lack of interest in routine activities (depression), apprehensions, physiological changes (anxiety), and tension- frustration (stress).^2^,^3^ Identification and comorbidity of these symptoms has been given due attention not only in the local context^2^ but also across the continents.^3^ Though, past studies^2^,^3^ along with many others have explored the two dimensional comorbidity of depression, anxiety and stress (e.g., depression and anxiety, depression and stress and anxiety and stress), yet unfortunately three dimensional (triadic) comorbidity remained unidentified and consequently untreated. Previously, a strong link has been found in depression, anxiety and stress symptoms in clinical sample of fibromyalgia patients^4^ as well as normative sample of students.^5^ Though these studies lacked triadic comorbidity but provide reasoning for estimation of three directional comorbidity of these symptoms; directing toward the presence of one dimension (e.g., depression) at different levels of other two (e.g., anxiety and stress). So far, no such studies in clinical as well as non-clinical samples have been conducted to examine the coexistence of mental health related symptoms from three aspects.

As MHPs face disadvantaged circumstances and mental health adversaries, therefore more vulnerable to have exposure of mental fatigue and emotional exhaustion, also called psychological burnout.^6^ Psychological burnout is described as emotional exhaustion, fatigue and damage to mental health in three contexts i.e., person (personal/self) related, client related and work/workplace related. Furthermore, the comorbidity of burnout at different levels e.g., moderate to low in clinical samples varies from 20% to 59% across the samples from Europe and Australia^7^ while measuring correlations rather than comorbidity. Further, how various demonstrations of burnout (e.g., person, client, and work related) at different levels of mental health symptoms prevail differently remained unexplored. While, addressing link between burnout and depression, anxiety and stress, few studies^8^,^9^ have examined traumatic stress widely but general/ routine life stress insufficiently. However, in relation to depression and anxiety symptoms, burnout has been generally reported as social withdrawal, negative thoughts, self-effacement and fatigue under symptoms of anxiety or depression rather than independent or distinct construct.^10^

Keeping in view current trends in psychiatric research and literature guidelines, the current study targeted to examine (i) triadic comorbidity of depression, anxiety and stress symptoms (ii) inter comorbidity of psychological burnout at different levels, and (iii) comorbidity of depression, anxiety and stress symptoms with psychological burnout.

## METHODS

A correlational study of 157 MHPs was conducted at three hospitals of Islamabad/ Rawalpindi (Holy Family Hospital=57, District Headquarter Hospital=60, and Benazir Bhutto Hospital=40) from June 2018 to September 2018. The sample size was determined by performing Raosoft website’s calculations, with 5% margin of error, 50% response distribution and 95% confidence interval. To control the extraneous factors, 157 doctors were selected through random sample technique, with minimum six months of service in the same hospitals were recruited. They were recruited through non-probability purposive sampling technique. Doctors serving for lesser duration, screened out or diagnosed for any mental or medical health issue, taking any counseling or therapeutic treatment, any history of illness or with any other comorbid conditions (e.g., hypertensions, chronic pain, diabetes etc) were excluded from the sample. Self-administered questionnaires e.g. a Demographic Questionnaire, Depression, Anxiety and Stress Scale (DASS)^11^ and Copenhagen Burnout Inventory (CBI)^12^ were used to assess mental health and psychological burnout respectively. The demographic questionnaire comprised of information about participants’ age, gender, placement and specialization. DASS is 21 items self-report measure of depression (*n=7*), anxiety (*n=7*) and stress (*n=7*) symptoms quantifying scores into normal, mild, moderate, severe, and extremely severe. CPI is 29 items self-report measure of burnout assessing burnout in three dimensions; work related *(n=7)*, client related *(n=6)* and personal related *(n=6)*. High scores are indicator of high level of burnout. A well proportionate sample of male (40%) and female (60%) doctors from general ward (*n*=64), emergency (*n*=60) and OPD (*n*=33) specialized in different areas (e.g., general medicine/ surgery, psychiatry, pediatrics, gynea, ENT etc,) participated in the current study.

Formal approval was obtained from Ethical Review Board of COMSATS University Islamabad, Lahore (May 1^st^ 2018) and written informed consent was taken from the participants. As it was one to one administration, response rate of study was high (i.e., 94%). Data was analyzed by using descriptive (mean and frequencies) as well as inferential statistics (Chi square test of association) by using SPSS version 24. In the first step, missing values analysis was performed by “replace with mean” method in SPSS. Triadic comorbidity was assessed through cross-tabulation. In addition to, Chi square test of association, psychological burnout (e.g., person, client and work related) were also cross-tabulated with mental health problems (depression, anxiety, and stress).

## RESULTS

Obtained data from 157 MHPs was well distributed in male and female (40% and 60% respectively). The triadic comorbidity of mental health symptoms [depression (5) × anxiety (5) stress (5)] was calculated through frequencies and percentages (Table-I). In case of comorbidity of normal level of stress, 26% (*n*=41) of the study sample demonstrated normal level of anxiety at normal level of depression. Whereas, no such comorbidity was observed at mild levels of depression, anxiety and stress. At moderate level of stress, 3% (n=5) of the participants showed moderated level of anxiety at the moderate level of depression. The triadic comorbidity for severe levels of anxiety, depression and stress symptoms was found as 1% (*n*=2). In context of excessive severe, 9% (*n*=12) of the doctors reporting excessive severe stress also reported excessive severe anxiety at excessive severe level of depression. Along with inter comorbidity of mental health problems, comorbidity for psychological burnout was also estimated. Results revealed that 4% (*n*=8) of the participants demonstrating severe level of client related burnout also reported sever work related and person related burnout. Whereas, in case of person related and work related, comorbidity at severe level was also observed as 8% (*n*=12) (Table-II). In addition to cross-tabulation, chi square test of association was also calculated to examine the cross comorbidity at five levels (normal, mild, moderate, severe and excessive severe) of depression, anxiety and stress symptoms across three (mild, moderate and severe) levels of psychological burnout (Table-III).

**Table I:** Triadic Comorbidity of Depression, Anxiety and Stress in Doctors.

Stress	Depression	Anxiety					

		Normal	Mild	Moderate	Severe	Ex. Severe	Total

		f(%)	f(%)	f(%)	f(%)	f(%)	f(%)
Normal	Normal	41(26%)	9(6%)	12(8%)	3(2%)	--	65(42%)
	Mild	2(1%)	4(3%)	11(6%)	3(2%)	--	20(13%)
	Moderate	2(1%)	4(3%)	3(2%)	--	--	9(6%)
	Severe	--	--	--	--	--	--
	Ex. Severe	--	--	--	--	--	--
	Total	45(28%)	17(11%)	26(16%)	6(4%)	--	94(61%)
Mild	Normal	--	1(1%)	--	--	--	1(1%)
	Mild	2(1%)	--	2(1%)	1(1%)	--	5(3%)
	Moderate	--	--	5(2%)	2(1%)	1(1%)	8(5%)
	Severe	1(1%)	--	--	--	--	2(1%)
	Ex. Severe	--	--	2(1%)	--	--	2(1%)
	Total	3(2%)	1(1%)	9(4%)	3(2%)	2(1%)	18(11%)
Moderate	Normal	--	--	--	--	--	--
	Mild	--	--	4(2%)	2(1%)	1(1%)	7(4%)
	Moderate	--	1(1%)	5(3%)	3(2%)	1(1%)	10(7%)
	Severe	--	--	3(2%)	2(1%)	4(3%)	9(6%)
	Ex. Severe	--	--	--	--	--	--
	Total	--	1(1%)	12(7%)	7(4%)	6(5%)	26(17%)
Severe	Normal	--	--	--	--	--	--
	Mild	--	--	--	2(1%)	3(2%)	5(3%)
	Moderate	--	--	--	--	5(3%)	5(3%)
	Severe	--	--	--	2(1%)	8(5%)	10(6%)
	Ex. Severe	--	--	1(1%)	-	8(5%)	10(6%)
	Total	--	--	1(1%)	5(2%)	24(15%)	29(18%)
Ex. Severe	Normal	41(27%)	10(6%)	12(8%)	3(2%)	--	66(42%)
	Mild	3(2%)	4(3%)	17(11%)	7(3%)	1(1%)	32(20%)
	Moderate	2(1%)	5(3%)	13(8%)	4(3%)	3(2%)	27(17%)
	Severe	2(1%)	--	4(2%)	2(2%)	7(3%)	15(10%)
	Ex. Severe	--	--	2(1%)	3(2%)	12(9%)	17(11%)
	Total	48(31%)	19(12)	48(30%)	19(12%)	23(15%)	157(100%)

Ex.= excessive severe.

**Table II:** Inter Comorbidity of Psychological Burnout.

Measures		Client Related Burnout			χ	Person Related Burnout			χ
	
		Mild	Moderate	Severe		Mild	Moderate	Severe	
	
		f(%)	f(%)	f(%)		f(%)	f(%)	f(%)	
Work Related Burnout	Mild	26(17%)	35(22%)	0	29.21***	52(33%)	15(9%)	0	90.82***
	Moderate	9(6%)	62(39%)	4(3%)		9(6%)	50(31%)	10(7%)	
	Severe	0	13(9%)	8(4%)		0	9(6%)	12(8%)	
Person Related Burnout	Mild	28(18%)	39(25%)	0	41.52***				
	Moderate	7(4%)	58(37%)	4(3%)					
	Severe	0	13(9%)	8(4%)					

***Note:***

*** =p<.0001

**Table III:** Association of Mental Health Problems with Psychological Burnout.

Measures	Persona Related Burnout			χ	Client Related Burnout			χ	Work Related Burnout			χ	Overall Burnout			χ
			
	Mild	Moderate	Severe		Mild	Moderate	Severe		Mild	Moderate	Severe		Mild	Moderate	Severe	
			
	f(%)	f(%)	f(%)		f(%)	f(%)	f(%)		f(%)	f(%)	f(%)		f(%)	f(%)	f(%)	
*Depression*																
Normal	37 (24%)	24 (15%)	3 (2%)	28.35***	21 (13%)	42 (27%)	2 (1%)	16.08*	37 (24%)	27 (16%)	22 (1%)	29.74***	28 (18%)	34 (22%)	--	31.28***
Mild	12 (8%)	19 (12%)	1 (2%)		4 (2%)	26 (16%)	2 (1%)		11 (7%)	18 (11%)	3 (2%)		9 (6%)	22 (14%)	2 (1%)	
Moderate	12 (8%)	10 (6%)	5 (3%)		8 (5%)	17 (9%)	2 (1%)		8 (5%)	16 (10%)	3 (2%)		8 (5%	16 (10%)	3 (2%)	
Severe	2 (1%)	11 (7%)	3 (2%)		1 (2%)	15 (10%)	--		3 (2%)	8 (5%)	5 (3%)		2 (1%)	12 (8%)	2 (1%)	
Ex. Severe	5 (3%)	5 (3%)	8 (5%)		2 (1%)	11 (7%)	4 (2%)		3 (2%)	6 (3%)	7 (4%)		2 (1%)	7 (4%)	10 (6%)	
Anxiety																
Normal	27 (17%)	17 (9%)	2 (1%)	30.63***	15 (10%)	31 (20%)	--	10.11	25 (16%)	20 (13%)	2 (1%)	13.07	21 (13%)	23 (15%)	2 (1%)	23.03**
Mild	11 (7%)	7 (4%)	1 (1%)		5 (3%)	13 (8%)	2 (1%)		10 (6%)	7 (4%)	2 (1%)		8 (5%)	10 (6%)	--	
Moderate	14 (9%)	31 (20%)	2 (1%)		11 (7%)	33 (21%)	4 (2%)		15 (10%)	27 (17%)	6 (3%)		9 (6%)	36 (23%)	2 (1%)	
Severe	7 (4%)	6 (4%)	6 (4%)		2 (1%)	16 (10%)	--		6 (3%)	10 (6%)	3 (2%)		6 (3%)	10 (6%)	3 (2%)	
Ex. Severe	9 (6%)	8 (5%)	9 (6%)		3 (2%)	18 (11%)	4 (2%)		6 (3%)	11 (7%)	7 (4%)		5 (3%)	12 (7%)	10 (6%)	
Stress																
Normal	49 (31%)	40 (25%)	3 (2%)	32.79***	27 (17%)	62 (39%)	4 (2%)	13.65	46 (29%)	43 (27%)	5 (3%)	35.60***	36 (23%)	53 (34%)	2 (1%)	38.81***
Mild	7 (4%)	8 (5%)	2 (1%)		5 (3%)	12 (8%)	--		6 (3%)	9 (6%)	2 (1%)		5 (3%)	12 (7%)	--	
Moderate	7 (4%)	15 (9%)	4 (2%)		2 (1%)	22 (14%)	2 (1%)		8 (5%)	14 (9%)	4 (2%)		6 (3%)	16 (10%)	4 (2%)	
Severe	3 (2%)	3 (2%)	4 (2%)		--	9 (6%)	1 (1%)		--	7 (4%)	2 (1%)		1 (1%)	7 (4%)	2 (1%)	
Ex. Severe	2 (1%)	3 (2%)	7 (4%)		2 (1%)	6 (4%)	3 (2%)		2 (1%)	2 (1%)	7 (4%)		1 (1%)	3 (2%)	9 (6%)	

Ex.= excessive sever

Table-III shows significant association of person related burnout with depression (*χ^2^=28.35, p<.0001*), anxiety (*χ^2^= 30.63, p<.0001*) and stress (*χ^2^= 32.79, p<.0001*), client related burnout with depression (*χ^2^=16.08, p<.05*) only, and work related burnout with depression (*χ^2^=29.74, p<.0001*) and stress (*χ^2^= 35.60, p<.0001*). Further, 5% (*n*=8) participants demonstrating excessive severe level of depression also exhibited severe level of person related burnout, 2% (*n*=4) of participants with excessive level of depression reported severe level of client related burnout, and 4% of the sample illustrating excessive severe depression indicated severe level of work related burnout. In case of anxiety symptoms at excessive severe level, 6% (*n*=9) of the study participants also revealed severe level of person related burnout, 2% (*n*=4) reported sever level of client related burnout, and 4% (*n*=7) demonstrated severe level of work related burnout. While assessing stress at excessive level, 4% (*n*=7) revealed severe level of person related burnout, 2% (n=3) reported sever level of client related burnout, and 4% (*n*=7) showed severe level of work related burnout.

Along with association of partial burnout (person, client and work related) with depression anxiety and stress, cumulative psychological burnout was also examined. The findings indicated significant association of depression (*χ^2^=31.28, p<.0001*), anxiety (*χ^2^= 23.03, p<.0001*) and stress (*χ^2^=38.81, p<.0001*) with psychological burnout. Further, findings indicated that 6% (*n*=10) of the study participants reporting excessive severe depression also reported sever burnout. Same (6%) comorbidity was observed in case of anxiety and stress.

## DISSCUSSION

To assess the comorbidity of mental health problem, and psychological burnout, a questionnaire based correlational study was carried out. The link between psychological burnout and mental health problems was also evaluated by using Chi square test of association. The current study demonstrated significant triadic comorbidity of depression, anxiety and stress symptoms which is unique finding but partially supplementing many previous observations^2^,^3^,^5^ illustrating significance two dimensional comorbidity of these symptoms. High co-existence of mental health problems in MHPs could be explained in the light of a psychological model^13^ focusing on the major role of cognitive evaluations between perception of an event and its reaction to it. Moreover, as MHPs have direct and frequent exposure of patients’ illness and also work in diverse but stressful settings (e.g., ward, OPD and emergency), therefore likely to be more vulnerable of manifesting symptoms of depression, anxiety and stress.

The present research finding also indicated significant coexistence of different dimensions of burnout particularly work related with person related at severe level supporting the previous observations directing toward the high degree of psychological and physical exhaustion and fatigue experienced by a person.^12^ This could be due to engagement of some personal characteristics found as common elements in these two scenarios (work related and person related) e.g., subjective perception, personal characteristics, workplace environment etc.^12^ Findings from this research also revealed significant association of mental health problems and psychological burnout which are incongruent with previous reports documenting a strong positive link between depression, anxiety and stress symptom and burnout across clinical sample of depressive patients^14^ and para medical staff^6^explaining 25% to 42% poor mental health due to psychological burnout. These findings further emphasized that depersonalization and emotional exhaustion likely to induce mental health problems.

When partial outing the independent effect of all three dimensions of psychological burnout, interesting results were seen. Depression and stress symptoms were significantly linked with all three dimensions of psychological burnout (i.e., client, person and work related), whereas anxiety symptoms were associated only with person related psychological burnout. Kerasidou and Horn’s theory of empathy explained this link quiet interestingly^15^ postulating that doctors are generally empathic toward their clients and workplace which brings concerns in them. Empathy being a very distinguished personal characteristic of an individual brings emotional exhaustion and over-concerned attitude while exerted more than needed. This may consequently induce apprehensions and anxiety symptoms in MHPs which may overrule the other form of burnouts (e.g., client and work related).^16^

The current study findings illustrated significant links between mental health problems and psychological burnout in MHPs. These findings are justified as that being a part of stressful field of medicine, MHPs are overburdened especially in the developing countries such as Pakistan where job demands are not sufficiently met due to a huge discrepancy between workload and work force. Consequently, they experience acute and chronic work pressure, emotional exhaustion and loss of energy initially and symptoms of depression, anxiety and stress in the long term.^16^ In addition to that, as psychological burnout in MHPs plays a significant role in inducing poor mental health symptoms in them, therefore its essential to conduct effective counseling/ therapeutic sessions for MHPs in order to minimize psychological burnout.

### Limitations of the study

Triadic co-existence of depression, anxiety and stress is unique and salient finding of current study. Though, this study signifies the importance of psychological burnout in relation to mental health issues, yet comes up with certain limitations. This correlational study explained a link between psychological burnout and mental health issues but failed to draw a causal inference which limits the interpretations in the broader context. A longitudinal research with same parameters may provide causal inferences. Further, data was collected from urban areas of Islamabad/ Rawalpindi which limits the generalizability of the findings to the exposure of stressful situations MHPs face in rural areas. Moreover, by controlling the confounding factors of subjects (e.g., age, gender, education, and work experience) and work related dimensions (e.g., placement, working hours, facilities), more rigorous effect of psychological burnout on mental health could be assessed. In extension to that dose-response relationship in context of work related stressors and mental and physical health complaints could be designed.

### Authors Contribution:

**FA:** conceived, designed and did statistical analysis, editing of manuscript and is responsible for **integrity of research**.

**HA** **and SA:** did data collection and manuscript writing.

**MS** **and AM:** did review and final approval of manuscript.
